# Timing of Naldemedine Initiation and Occurrence of Diarrhea in Patients Receiving Strong Opioid Analgesics: A Retrospective Study

**DOI:** 10.3390/pharmacy13020047

**Published:** 2025-03-21

**Authors:** Takuma Matsumoto, Takuya Mura, Tsubasa Wada, Yuki Tsugo, Naoko Mukai, Terutaka Hamaoka, Shuji Horita, Yasushi Semba, Shinichi Watanabe

**Affiliations:** 1 Department of Hospital Pharmacy, NHO Shikoku Cancer Center, 160 Minamiumemoto-machi-kou, Matsuyama 791-0280, Ehime, Japan; wada.tsubasa.sg@mail.hosp.go.jp (T.W.); tsugo.yuuki.aw@mail.hosp.go.jp (Y.T.); futagami.naoko.nz@mail.hosp.go.jp (N.M.); horita.shuji.qh@mail.hosp.go.jp (S.H.); semba.yasushi.jw@mail.hosp.go.jp (Y.S.); 2 Department of Hospital Pharmacy, NHO Iwakuni Clinical Center, 1-1-1 Atago-cho, Iwakuni 740-8510, Yamaguchi, Japan; mura.takuya.yh@mail.hosp.go.jp; 3 Department of Hospital Pharmacy, NHO Fukuyama Medical Center, 4-14-17 Okinogami-cho, Fukuyama 720-8520, Hiroshima, Japan; hamaoka.terutaka.eu@mail.hosp.go.jp; 4 Department of Clinical Pharmacy, College of Pharmaceutical Sciences, Matsuyama University, 4-2 Bunkyo-cho, Matsuyama 790-8578, Ehime, Japan; swatanab@g.matsuyama-u.ac.jp

**Keywords:** naldemedine, diarrhea, strong opioid analgesics

## Abstract

Naldemedine, a peripherally acting μ-opioid receptor antagonist, is used to treat opioid-induced constipation (OIC). However, it causes diarrhea as an adverse effect. This retrospective study aimed to investigate whether the occurrence of diarrhea was dependent on the timing of naldemedine treatment initiation. Inpatients who were initially treated with naldemedine at the Department of Respiratory Medicine, NHO Iwakuni Medical Center, Japan, between 1 December 2017 and 31 March 2021 were included in this study and divided into the simultaneous combination group, in which naldemedine was introduced at the same time as strong opioid analgesics, and the non-simultaneous combination group, in which naldemedine was introduced after the initiation of treatment with strong opioid analgesics. This study included 45 patients, 15 (33.3%) of whom developed diarrhea. Among the patients in the simultaneous combination group and non-simultaneous combination group, diarrhea occurred in 2 (11.1%) and 13 (48.1%) patients, respectively. Multivariate logistic regression analysis revealed that the delayed introduction of naldemedine was significantly associated with the development of diarrhea (odds ratio: 6.68, 95% confidence interval: 1.220–36.700, *p* = 0.028). Our analysis reveals that the simultaneous administration of naldemedine and oxycodone may prevent the development of diarrhea associated with naldemedine use for OIC.

## 1. Introduction

Opioid analgesics, which are widely used to relieve moderate-to-severe cancer-associated pain, act primarily by stimulating μ-opioid receptors in the central nervous system [1,2,3]. Typical adverse events (AEs) associated with opioid analgesic use include constipation, nausea, vomiting, and drowsiness [4]. Of these, nausea, vomiting, and drowsiness often occur when treatment is initiated or when the dose is increased; however, they diminish or resolve within days [5]. In contrast, opioid-induced constipation (OIC) occurs as a result of μ-opioid receptor stimulation in the intestinal tract, which inhibits intestinal peristalsis and causes persistent constipation [6,7]. OIC has been reported to occur in 39–85.7% of patients receiving long-term opioid treatment [8,9,10,11], and a survey of hospitalized patients with cancer in Japan revealed that approximately 40% of the patients experienced constipation [12]. OIC impacts the quality of life (QOL) of patients and can also lead to a reduction or discontinuation of opioid analgesic use, thus increasing pain levels [13,14,15].

The Japanese Society for Palliative Medicine and the American Gastroenterological Association (AGA) recommend conventional laxatives as first-line therapeutics for patients with OIC [5,16]. However, it has been reported that laxatives are ineffective in the treatment of OIC and instead increase AEs in patients, leading to a further reduction in QOL [17,18]. The AGA guidelines recommend the use of naldemedine as a second-line therapeutic if laxative treatment proves unsatisfactory.

Naldemedine is a peripherally acting μ-opioid receptor antagonist that minimizes the effects of exogenous opioids at peripheral μ-opioid receptors, including those in the gastrointestinal tract, without affecting receptors in the central nervous system responsible for the analgesic effects of opioids [19,20,21,22]. The most common AE associated with naldemedine is diarrhea, and 5% of patients who develop diarrhea discontinue naldemedine treatment [23]. Administration of naldemedine early after the initiation of treatment with opioid analgesics has been reported to decrease the incidence of diarrhea [24,25].

Although naldemedine is not currently recommended in Japan as a first-line treatment for OIC, Japanese prospective observational studies and randomized controlled trials have indicated its effectiveness as a prophylactic treatment for OIC [26,27]. However, few studies have reported the occurrence of diarrhea during prophylactic administration of naldemedine for OIC in clinical practice. Therefore, this study aimed to compare the incidence of diarrhea after concurrent and non-concurrent administration of naldemedine and opioid analgesics.

## 2. Materials and Methods

### 2.1. Patient Selection

**Figure 1:**
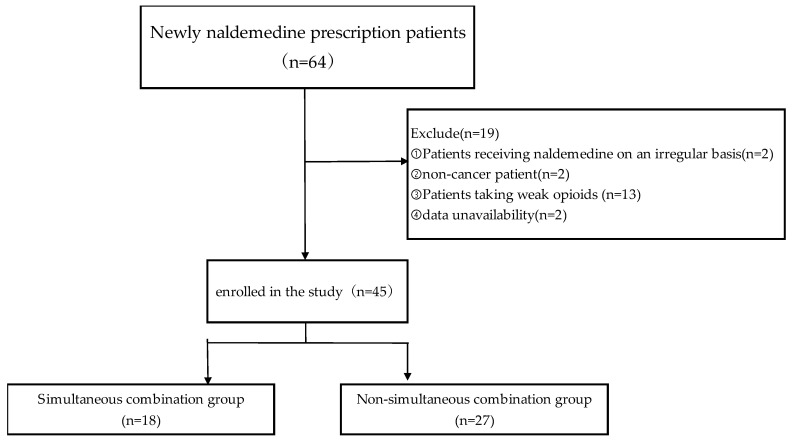
Flow chart for patient selection.

This retrospective study included data collected from electronic medical records on 64 patients with lung cancer who were treated with naldemedine upon admission to the Department of Respiratory Medicine, NHO Iwakuni Medical Center, between 1 December 2017 and 31 March 2021. Patients who received naldemedine on an irregular basis, did not have cancer, were taking weak opioid analgesics, already exhibited diarrhea at the start of naldemedine administration, or had missing data were excluded from the study. (Figure 1). The patients were divided into two groups, depending on the timing of naldemedine administration: simultaneous combination, in which both agents were initiated at the same time, and non-simultaneous combination, in which naldemedine was introduced on or after the second day following the initiation of strong opioid analgesics.

Data regarding sex, age, height, weight, body surface area (BSA), performance status, suspected blood–brain barrier dysfunction, type of strong opioid, hematological parameters before naldemedine administration, and concomitant medications were collected. BSA was calculated using the Dubois formula: BSA [m^2^] = [body weight(kg)]^0.425^ × [height(cm)]^0.725^ × 0.007184 [28]. Blood–brain barrier dysfunction may be a result of brain tumors, dementia associated with acquired immunodeficiency syndrome, multiple sclerosis, or Alzheimer’s disease [29].

### 2.2. Ethics

This study was approved by the Ethics Committee of NHO Iwakuni Medical Center (Approval No. 0347) and conducted in accordance with the principles outlined in the Declaration of Helsinki. Data were collected from medical records in accordance with the Ethical Guidelines for Medical and Biological Research Involving Human Subjects in Japan. Information regarding the conduct of this study was disclosed on the website, which also provided an opportunity for patients to opt out of the study.

### 2.3. Evaluation of Diarrhea

Diarrhea was defined as “diarrhea” written on the medical record within 2 weeks of the initiation of naldemedine administration, as previously described [23]. Diarrhea severity was assessed as grades 1–4, using a Japanese version of the Common Terminology Criteria for Adverse Events, version 5.0, as translated by the Japanese Clinical Oncology Group.

### 2.4. Statistical Analysis

Patient characteristics were analyzed using Fisher’s exact probability test and the Mann–Whitney U test for categorical and continuous variables, respectively. Univariate logistic regression analyses were performed to evaluate the odds ratio, 95% confidence intervals, and *p* value of each potential confounding factor for diarrhea induced by naldemedine. Multivariate logistic regression analysis was performed to exclude confounding factors. Multivariate logistic regression analysis was performed using sex and oxycodone as explanatory variables; these potential confounding factors were selected after reviewing the relevant literature. Results are expressed as odds ratios with 95% confidence intervals, and differences were considered statistically significant with a two-sided *p* value ≤ 0.05. Statistical analyses were performed using EZR software, version 1.66 [30].

## 3. Results

### 3.1. Patients

The patient selection process is summarized in Figure 1. Of the 64 inpatients screened, 19 patients were excluded from the study: 2 received naldemedine on an irregular basis, 2 did not have cancer, 2 had missing data, and 13 were administered weak opioid analgesics. Finally, 45 patients were included in the analysis, with 18 patients in the simultaneous combination group and 27 in the non-simultaneous combination group.

The baseline patient characteristics are summarized in Table 1. The median age of the patients was 71 years, and 33 patients (73%) were men. The performance status of 28 patients (62%) was 1. Concomitant medications included drugs that may be risk factors for diarrhea (proton pump inhibitor, histamine H_2_ receptor antagonist, non-steroidal anti-inflammatory drugs, selective serotonin reuptake inhibitors, aspirin, anticholinergics, digestive stimulant, and constipation medication) [31]; the CYP3A4 inducers, aprepitant and dexamethasone; and the P-glycoprotein inhibitors, suvorexant and tolvaptan. The median number of three consecutive days of spontaneous defecations prior to naldemedine administration was 1 in both the simultaneous and non-simultaneous combination groups. The median duration of opioid therapy prior to naldemedine administration was 3 days in the non-simultaneous combination group. The opioid analgesics administered were morphine or oxycodone in 35 (77.8%) of the 45 patients. The incidence of diarrhea was higher in the group that was administered oxycodone. Other baseline characteristics did not significantly differ between the simultaneous combination and the non-simultaneous combination groups.

### 3.2. Incidence and Severity of Diarrhea

We summarized the incidence and severity of diarrhea in each group (Table 2). Diarrhea occurred in 15 (33.3%) of the 45 patients. Grade 1 diarrhea was experienced by 14 patients (93.3%), and grade 2 diarrhea was experienced by 1 (6.7%) patient; no cases of grade 3 or 4 diarrhea were observed. The incidence of diarrhea was significantly lower in the simultaneous combination group (n = 2; 11.1%) than in the non-simultaneous combination group (n = 13; 48.1%) (95% confidence interval: 1.269–75.679, *p* = 0.011).

### 3.3. Factors Influencing the Development of Diarrhea

Univariate analysis suggested that the non-simultaneous administration of naldemedine was associated with the development of diarrhea (odds ratio: 7.43; 95% confidence interval: 1.42–38.80, *p* = 0.017) (Table 3). The results of the multivariate analysis identified the non-simultaneous introduction of naldemedine as an independent risk factor for the development of diarrhea (odds ratio: 6.68; 95% confidence interval: 1.220–36.700, *p* = 0.028).

## 4. Discussion

This retrospective study of the occurrence of diarrhea following naldemedine administration in patients with lung cancer assessed the impact of the timing of naldemedine treatment initiation. The results showed that diarrhea occurred in 11.1% of patients in whom naldemedine and strong opioid analgesics were introduced simultaneously. This is consistent with prior studies that reported the development of diarrhea in 8–10% of cases [27]. However, the present study also showed that diarrhea occurred in 48.14% of patients in whom the introduction of naldemedine was delayed. This is consistent with the findings of Japanese clinical trials in patients with cancer and OIC, which reported the development of diarrhea in 19.6–39.7% of patients [21,23]. Longer durations of opioid administration before initiating naldemedine treatment and initiating naldemedine treatment 8 days after the initiation of opioid analgesia have been reported as risk factors for diarrhea, suggesting the benefits of initiating naldemedine and opioid analgesics simultaneously [24,25,32]. However, it has also been reported that the prophylactic effect of naldemedine against OIC is low for oxycodone [33].

In this study, we also considered the concomitant use of constipation medications, sex, and intravenous anticancer drugs as potential confounding factors in the multivariate analysis [32]. Delayed naldemedine introduction was positively associated with the occurrence of diarrhea, even after adjusting for these factors. However, more patients in the non-simultaneous group were taking constipation medications. Although there was no significant difference, the patient population with abnormal bowel movements may be more likely to be included in the non-simultaneous group because they are taking constipation medications.

OIC is mainly caused by the binding of exogenous opioids and peripheral μ-opioid receptors in the submucosal plexus and mesenteric plexus of the enteric nervous system of the gastrointestinal tract. This causes changes in the neural output from the enteric nervous system, resulting in a decrease in intestinal motility, a decrease in intestinal fluid secretion, and an increase in intestinal fluid absorption. Naldemedine restores gastrointestinal function by inhibiting the action of opioids at peripheral μ-opioid receptors in the gastrointestinal tract, which may thereby promote the development of diarrhea [22,34]. Naldemedine is metabolized by CYP3A4 and is a substrate for P-glycoprotein. Concomitant use of CYP3A4 inhibitors or P-glycoprotein may therefore increase blood levels of naldemedine and the incidence of diarrhea, and is thus a potential confounding factor. This was particularly noticeable in the group being administered oxycodone.

Although this study showed that the incidence of diarrhea was reduced when naldemedine and strong opioid analgesics were introduced simultaneously, careful consideration is required before adopting this strategy. Naldemedine is not currently recommended as a first-line treatment for OIC, and the economic burden of administering naldemedine to patients without OIC should also be taken into account [35,36].

However, clinical practice does not always adhere to guidelines, and it has previously been reported that 12.2% of patients were simultaneously administered laxatives and naldemedine [37]. Considering the high prevalence of OIC and its impact on patient QOL, early administration of naldemedine may be a valid treatment option [8,9,10,11,12,17,18].

This study exhibited several limitations. First, this was a retrospective study involving a small number of patients conducted at a single institution, based on data obtained from medical records, from which some information may have been missing or recorded inconsistently. Second, owing to the retrospective nature of the study, we cannot exclude the potential influence of other unknown confounding factors, such as physical activity and dietary intake, on the development of diarrhea. Third, because this study focused on AEs, we did not examine treatment efficacy. Further prospective studies are therefore needed to confirm our findings and address the clinical questions raised by this study.

## 5. Conclusions

In conclusion, although the results are from a retrospective study with a small sample size, they indicate that the simultaneous administration of naldemedine and oxycodone may prevent the development of diarrhea.

## Figures and Tables

**Table 1:** Patient characteristics.

Characteristic	All Patients (n = 45)	Simultaneous Combination (n = 18)	Non-Simultaneous Combination (n = 27)	*p* Value
Sex (female)	12 (26.7%)	7 (38.8%)	5 (18.5%)	0.175 ^(a)^
Age (years)	71 [65–80]	71 [63–77]	72 [66–81]	0.215 ^(b)^
Height (cm)	162.2 [155.5–168.5]	162 [154.5–166.6]	164.4 [156.9–169.3]	0.424 ^(b)^
Weight (kg)	53.9 [44.2–64]	55.1 [45.3–64.1]	51.7 [45.1–59.8]	0.516 ^(b)^
BSA (m^2^)	1.569 [1.402–1.688]	1.599 [1.415–1.683]	1.535 [1.419–1.694]	0.853 ^(b)^
Performance status (2–3)	17 (37.8%)	5 (27.8%)	12 (44.4%)	0.315 ^(a)^
Serum creatinine (mg/dL)	0.75 [0.64–0.95]	0.79 [0.72–0.95]	0.71 [0.61–0.93]	0.190 ^(b)^
ALB (g/L)	2.8 [2.2–3.4]	3.25 [2.5–3.75]	2.7 [2.15–3.1]	0.053 ^(b)^
AST (IU/L)	24 [18–35]	21.5 [18–38.8]	24 [18.5–33]	0.954 ^(b)^
ALT (IU/L)	16 [12–32]	18 [10–52.3]	15 [13–21.5]	0.618 ^(b)^
eGFR (mL/min/1.73 m^2^)	75 [55–90]	65 [48.5–76.8]	79 [60–96]	0.088 ^(b)^
Suspected blood–brain barrier dysfunction	0	0	0	
Duration of opioid therapy before naldemedine administration (days)	1 [0–5]	0	3 [1–8]	<0.001 ^(b)^
Opioid analgesics (regular dosing)				
Morphine	15 (33.3%)	7 (38.9%)	8 (29.6%)	0.538 ^(a)^
Oxycodone	20 (44.4%)	6 (33.3%)	14 (51.9%)	0.359 ^(a)^
Fentanyl	1 (2.2%)	0	1 (3.7%)	1.000 ^(a)^
Hydromorphone	9 (20.0%)	5 (27.8%)	4 (14.8%)	0.192 ^(a)^
Onset of diarrhea				
Morphine	4 (8.9%)	2 (11.1%)	2 (7.4%)	1.000 ^(a)^
Oxycodone	10 (22.2%)	0	10 (37.0%)	0.011 ^(a)^
Fentanyl	0	0	0	
Hydromorphone	1 (2.2%)	0	1 (3.7%)	0.444 ^(a)^
Median number of 3-day defecations prior to naldemedine (−3 to −1)	3 [1–5.5]	1 [0.25–2]	1 [1–3]	0.192 ^(a)^
Co-administrated drugs				
Drug with risk of constipation	38 (84.4%)	14 (77.8%)	24 (88.9%)	0.412 ^(a)^
PPI	28 (62.2%)	12 (66.7%)	16 (59.3%)	0.757 ^(a)^
H_2_-blocker	3 (6.7%)	0	3 (11.1%)	0.264 ^(a)^
NSAIDs	30 (66.7%)	10 (55.6%)	20 (74.1%)	0.218 ^(a)^
SSRI	1 (2.2%)	0	1 (3.7%)	1.000 ^(a)^
Aspirin	2 (4.4%)	0	2 (7.4%)	0.509 ^(a)^
Anticholinergics	0	0	0	
Digestive stimulant	7 (15.6%)	1 (5.6%)	6 (22.2%)	0.215 ^(a)^
Concomitant use of constipation medication (regular dosing)	30 (66.7%)	13 (72.2%)	17 (63.0%)	0.748 ^(a)^
Magnesium oxide	28 (62.2%)	13 (72.2%)	15 (55.6%)	0.351 ^(a)^
Lubiprostone	5 (11.1%)	3 (16.7%)	2 (7.4%)	0.375 ^(a)^
Linaclotide	1 (2.2%)	1 (5.6%)	0	0.400 ^(a)^
CYP3A4 inhibitor	0	0	0	
CYP3A4 inducer	22 (48.9%)	8 (44.4%)	14 (51.9%)	0.763 ^(a)^
P-glycoprotein inhibitor	4 (8.9%)	2 (11.1%)	2 (7.4%)	0.375 ^(a)^
Intravenous anticancer drug	28 (62.2%)	13 (72.2%)	15 (55.6%)	0.351 ^(a)^
Amrubicin	2 (4.4%)	2 (11.1%)	0	0.155 ^(a)^
Atezolizumab	2 (4.4%)	1 (5.6%)	1 (3.7%)	1.000 ^(a)^
Bevacizumab	2 (4.4%)	0	2 (7.4%)	0.509 ^(a)^
Carboplatin	3 (6.7%)	1 (5.6%)	2 (7.4%)	1.000 ^(a)^
Cisplatin	2 (4.4%)	0	2 (7.4%)	0.509 ^(a)^
Dacomitinib	1 (2.2%)	0	1 (3.7%)	1.000 ^(a)^
Docetaxel	1 (2.2%)	1 (5.6%)	0	0.400 ^(a)^
Gemcitabine	2 (4.4%)	0	2 (7.4%)	0.509 ^(a)^
Nivolumab	2 (4.4%)	1 (5.6%)	1 (3.7%)	1.000 ^(a)^
Osimertinib	1 (2.2%)	0	1 (3.7%)	1.000 ^(a)^
Paclitaxel	3 (6.7%)	2 (11.1%)	1 (3.7%)	0.555 ^(a)^
Pembrolizumab	5 (11.1%)	3 (16.7%)	2 (7.4%)	0.375 ^(a)^
Pemetrexed	6 (13.3%)	2 (11.1%)	4 (14.8%)	1.000 ^(a)^
Tegafur/Gimeracil/Oteracil potassium	4 (8.9%)	2 (11.1%)	2 (7.4%)	1.000 ^(a)^
Vinorelbine	1 (2.2%)	0	1 (3.7%)	1.000 ^(a)^

Data are expressed as median [interquartile range] or number (%). Statistical analysis was performed using ^(a)^ Fisher’s exact probability test or ^(b)^ the Mann–Whitney U test. ALB, albumin; ALT, alanine aminotransferase; AST, aspartate aminotransferase; BSA, body surface area; eGFR, estimated glomerular filtration rate; PPI, proton pump inhibitor.

**Table 2:** Onset and severity of diarrhea according to the timing of naldemedine administration.

Diarrhea	Simultaneous Combination (n = 18)	Non-Simultaneous Combination (n = 27)	95% CI	*p* Value
No diarrhea	16 (88.9%)	14 (51.9%)		
Diarrhea	2 (11.1%)	13 (48.1%)	1.269–75.679	0.011
Grade 1	2 (11.1%)	12 (44.4%)		
Grade 2	0 (0%)	1 (3.7%)		
Grade 3	0 (0%)	0 (0%)		
Grage 4	0 (0%)	0 (0%)		

Data are expressed as numbers (%). Diarrhea was evaluated using the Common Terminology Criteria for Adverse Events, version 5.0.

**Table 3:** Univariate and multivariate analysis of factors influencing the incidence of diarrhea.

Variable	Univariate Analysis			Multivariate Analysis		
	Odds Ratio	95% CI	*p* Value	Odds Ratio	95% CI	*p* Value
Non-simultaneous naldemedine administration	7.430	1.420–38.800	0.017	6.680	1.220–36.700	0.028
Sex	1.000	0.289–3.460	1.000	0.574	0.104–3.180	0.525
Oxycodone	4.000	1.070–14.900	0.038	3.530	0.857–14.500	0.080
Intravenous anticancer drug	0.868	0.243–3.100	0.828			
Concomitant use of constipation medication	1.000	0.269–3.720	1.000			

Logistic regression analysis was performed. CI, confidence interval.

## Data Availability

Data will be made available upon reasonable request, following the institutional applicable guidelines and approvals.

## Abbreviations

The following abbreviations are used in this manuscript:

AEs	adverse events
OIC	opioid-induced constipation
QOL	quality of life
AGA	American Gastroenterological Association
BSA	body surface area
